# (1,6,7,12-Tetra­aza­perylene-κ^2^ *N*,*N*′)bis­(4,4′,5,5′-tetra­methyl-2,2′-bipyridyl-κ^2^ *N*,*N*′)ruthenium(II) bis­(hexa­fluorido­phosphate) aceto­nitrile tris­olvate

**DOI:** 10.1107/S1600536814011969

**Published:** 2014-05-31

**Authors:** Thomas Brietzke, Daniel Kässler, Alexandra Kelling, Uwe Schilde, Hans-Jürgen Holdt

**Affiliations:** a Universität Potsdam, Institut für Chemie, Anorganische Chemie, Karl-Liebknecht-Strasse 24-25, D-14476 Potsdam, Germany

## Abstract

In the title compound, *rac*-[Ru(C_14_H_16_N_2_)_2_(C_16_H_8_N_4_)](PF_6_)_2_·3C_2_H_3_N, discrete dimers of complex cations, [Ru(tmbpy)_2_­tape]^2+^, of opposite chirality are formed (tmbpy = tetra­methyl­bipyridine; tape = tetraazaperylene), held together by π–π stacking inter­actions between the tetra­aza­perylene moieties with centroid–centroid distances in the range 3.563 (3)–3.837 (3) Å. These inter­actions exhibit a parallel displaced π–π stacking mode. Additional weak C—H⋯π-ring and C—H⋯N and C—H⋯F inter­actions are found, leading to a three-dimensional architecture. The Ru^II^ atom is coordinated in a distorted octa­hedral geometry. The counter-charge is provided by two hexa­fluorido­phosphate anions and the asymmetric unit is completed by three aceto­nitrile solvent mol­ecules of crystallization. Four F atoms of one PF_6_^−^ anion are disordered over three sets of sites with occupancies of 0.517 (3):0.244 (3):0.239 (3). Two aceto­nitrile solvent mol­ecules are highly disordered and their estimated scattering contribution was subtracted from the observed diffraction data using the SQUEEZE option in *PLATON* [Spek (2009 ▶). *Acta Cryst.* D**65**, 148–155].

## Related literature   

For related Ru^II^ complexes with tape and bpy-type ligands, see: Brietzke *et al.* (2012 ▶). For background to the alkaloid eilatin, see: Rudia *et al.* (1988 ▶). For Ru^II^ complexes including eilatin-type ligands, see: Gut *et al.* (2002 ▶); Bergman *et al.* (2004 ▶, 2005 ▶).
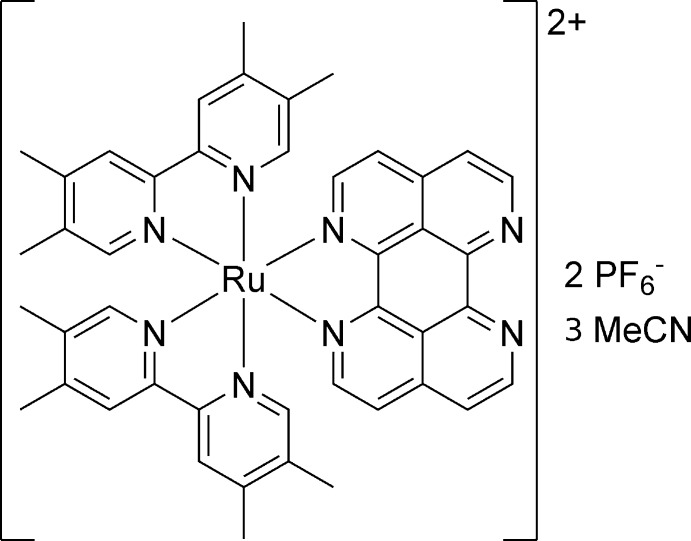


## Experimental   

### 

#### Crystal data  

[Ru(C_14_H_16_N_2_)_2_(C_16_H_8_N_4_)](PF_6_)_2_·3C_2_H_3_N
*M* *_r_* = 1195.01
Triclinic, 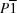

*a* = 12.7485 (5) Å
*b* = 13.6973 (7) Å
*c* = 17.3623 (9) Å
α = 105.786 (4)°
β = 92.858 (4)°
γ = 110.436 (3)°
*V* = 2698.3 (2) Å^3^
*Z* = 2
Mo *K*α radiation
μ = 0.44 mm^−1^
*T* = 210 K
0.55 × 0.40 × 0.25 mm

#### Data collection  

Stoe IPDS-2 diffractometer
Absorption correction: integration (*X-RED*; Stoe & Cie, 2011 ▶) *T*_min_ = 0.758, *T*_max_ = 0.955
17495 measured reflections
8918 independent reflections
6396 reflections with *I* > 2σ(*I*)
*R*_int_ = 0.044

#### Refinement  

*R*[*F* ^2^ > 2σ(*F* ^2^)] = 0.047
*wR*(*F* ^2^) = 0.124
*S* = 0.94
8918 reflections
743 parameters
363 restraints
H-atom parameters constrained
Δρ_max_ = 0.51 e Å^−3^
Δρ_min_ = −0.54 e Å^−3^

### 

Data collection: *X-AREA* (Stoe & Cie, 2011 ▶); cell refinement: *X-AREA*; data reduction: *X-RED* (Stoe & Cie, 2011 ▶); program(s) used to solve structure: *SHELXS97* (Sheldrick, 2008 ▶); program(s) used to refine structure: *SHELXL2013* (Sheldrick, 2008 ▶); molecular graphics: *DIAMOND* (Brandenburg, 2012 ▶) and *ORTEP-3 for Windows* (Farrugia, 2012 ▶); software used to prepare material for publication: *SHELXL2013* and *PLATON* (Spek, 2009 ▶).

## Supplementary Material

Crystal structure: contains datablock(s) global, I. DOI: 10.1107/S1600536814011969/tk5315sup1.cif

Structure factors: contains datablock(s) I. DOI: 10.1107/S1600536814011969/tk5315Isup2.hkl

CCDC reference: 1004679

Additional supporting information:  crystallographic information; 3D view; checkCIF report

## Figures and Tables

**Table 1:** Selected bond lengths (Å)

N1—Ru1	2.048 (3)
N4—Ru1	2.047 (3)
N5—Ru1	2.074 (3)
N6—Ru1	2.065 (3)
N7—Ru1	2.063 (3)
N8—Ru1	2.061 (3)

**Table 2:** Hydrogen-bond geometry (Å, °) *Cg*1, *Cg*2 and *Cg*3 denote the centroids of the N7/C31–C35, N8/C36–C40 and N6/C22–C26 rings, respectively.

*D*—H⋯*A*	*D*—H	H⋯*A*	*D*⋯*A*	*D*—H⋯*A*
C2—H2⋯F10^i^	0.93	2.61	3.402 (7)	143
C3—H3⋯F10^i^	0.93	2.50	3.318 (8)	147
C6—H6⋯F8^ii^	0.93	2.58	3.218 (8)	126
C8—H8⋯N7	0.93	2.65	3.166 (5)	116
C20—H20⋯F1*A* ^iii^	0.93	2.41	3.320 (6)	166
C20—H20⋯F5*C* ^iii^	0.93	2.64	3.411 (15)	141
C23—H23⋯F1*A* ^iii^	0.93	2.55	3.479 (5)	177
C26—H26⋯N8	0.93	2.59	3.138 (5)	118
C31—H31⋯F6*A*	0.93	2.63	3.461 (13)	150
C34—H34⋯F5*A* ^iv^	0.93	2.29	3.170 (8)	158
C34—H34⋯F6*B* ^iv^	0.93	2.30	3.129 (11)	148
C34—H34⋯F6*C* ^iv^	0.93	2.55	3.295 (19)	137
C45—H45*A*⋯F11^ii^	0.96	2.49	3.343 (12)	148
C45—H45*C*⋯F11	0.96	2.58	3.388 (15)	142
C47—H47*A*⋯N9^v^	0.96	2.66	3.452 (14)	140
C47—H47*B*⋯F5*B* ^iii^	0.96	2.55	3.343 (16)	140
C47—H47*B*⋯F5*C* ^iii^	0.96	2.59	3.293 (15)	131
C8—H8⋯*Cg*1	0.93	2.92	3.711 (5)	144
C26—H26⋯*Cg*2	0.93	2.90	3.708 (4)	146
C42—H42*A*⋯*Cg*3^vi^	0.96	2.79	3.339 (5)	117

Symmetry codes: (i) 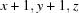; (ii) 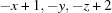; (iii) 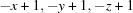; (iv) 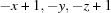; (v) 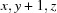; (vi) 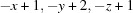.

**Fig. 1.**
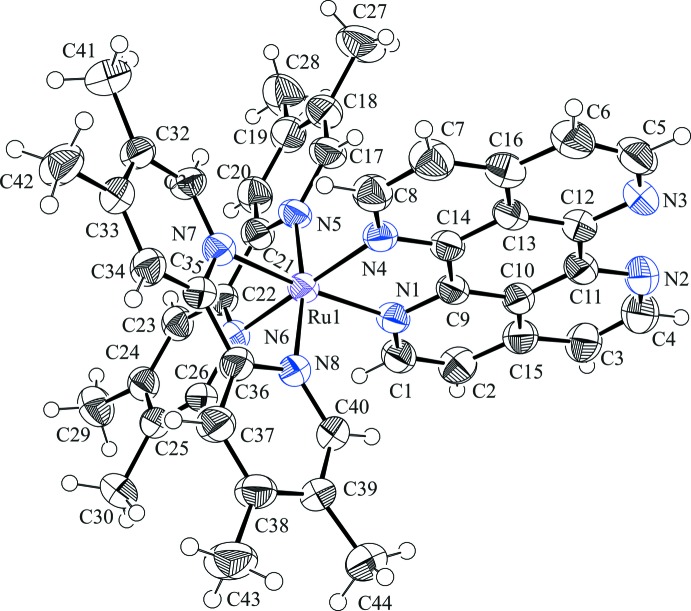
ORTEP drawing of Λ-[Ru(tmbpy)2(tape)]2+ in rac-[Ru(tmbpy)2(tape)](PF6)2·3MeCN with the atomic numbering scheme and 30% probability displacement ellipsoids. Anions and solvent molecules are omitted for clarity.

**Fig. 2.**
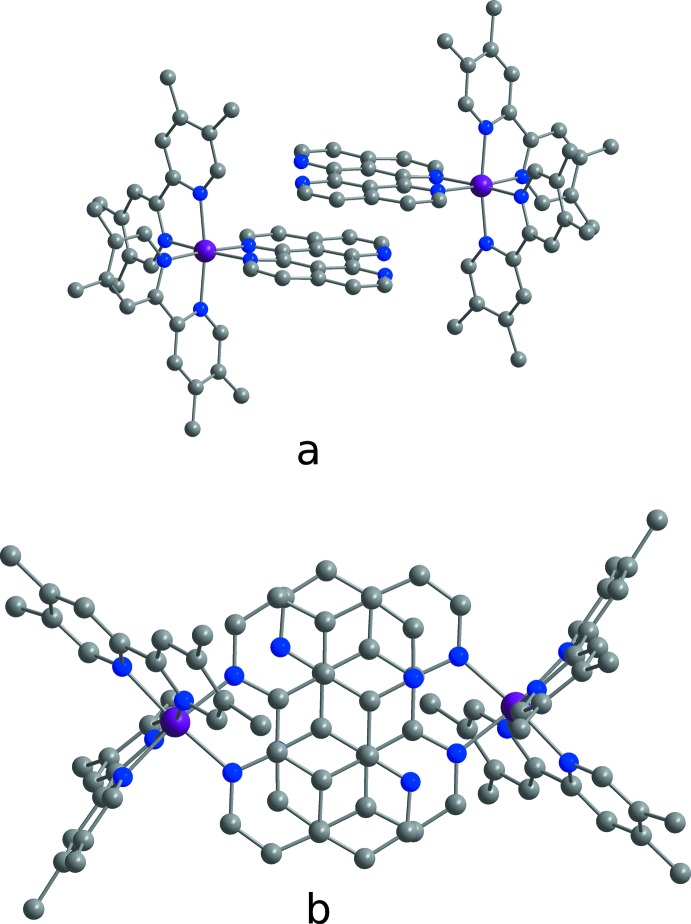
a: A side view of the dimer formed by Λ-[Ru(tmbpy)2(tape)]2+ and Δ-[Ru(tmbpy)2(tape)]2+ in rac-[Ru(tmbpy)2(tape)](PF6)2·3MeCN, featuring the stacking interactions via planar tape moieties. b: View along the normal of the tape-given r.m.s. plain, demonstrating the parallel-displaced π–π-stacking between the tape moieties. Anions, solvent molecules and hydrogen atoms are omitted for clarity.

**Fig. 3.**
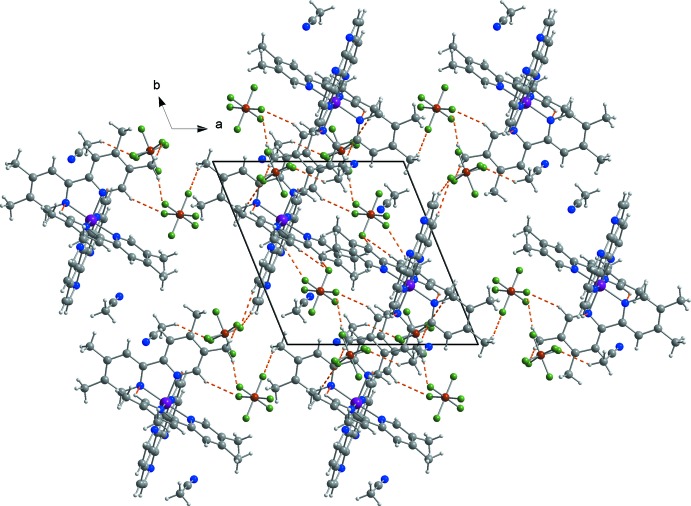
A packing diagram of the title compound is displayed along the c axis. Hydrogen bonds are shown as orange dashed lines.

## supplementary crystallographic information

### 1. Structural commentary

The ligand 1,6,7,12-tetra­aza­perylene (tape) is a *D*_2h_-symmetric bis­(*α*,*α'*-di­imine) ligand containing an extended π-heteroaromatic system. Tape is closely related to the well known ligand 2,2'-bi­pyrimidine (bpym). However, building supra­molecular structures by π–π stacking inter­actions is an additional feature for complexes containing terminal tape ligands. Such behavior was described for Ru^II^ and Os^II^ complexes of eilatin (dibenzo-[b,n]-1,6,7,12-tetra­aza­perylene) (Gut *et al.*, 2002), isoeilatin (dibenzo-[b,k]-1,6,7,12-tetra­aza­perylene) (Bergman *et al.*, 2005) and dibenzoeilatin (tetra­benzo-[b,e,k,n]-1,6,7,12-tetra­aza­perylene) (Bergman *et al.*, 2004). Eilatin, an alkaloid first isolated from a Red Sea tunicate (Rudia *et al.*, 1988), isoeilatin and dibenzoeilatin all have a tape core, mainly determining the electronic properties of these large surface ligands (Bergman *et al.*, 2005). The synthesis of tape, as uncoordinated ligand, was first published in 2012 by our workgroup (Brietzke *et al.*, 2012). In the same article, we compared UV-Vis absorption spectra, redox properties as well as structures for Ru^II^ complexes of the formula [Ru(L–L)tape]^2+^ (with L–L = phen, bpy, dmbpy (4,4'-di­methyl-2,2'-bi­pyridine), dtbbpy (4,4'-di-tert-butyl-2,2'-bi­pyridine) and tmbpy (4,4'5,5'-tetra­methyl-2,2'-bi­pyridine)). However, we could not present the structures of the Ru^II^ complexes with L–L = phen and tmpby in this first report, because there were no crystals suitable for X-ray diffraction. To fill one part of this gap, we present herein the structure of *rac*-[Ru(tmbpy)_2_tape](PF_6_)_2_·3MeCN, Fig. 1 & Table 1. The key feature in the crystal packing of this compound, and of the analogous complexes mentioned above, is the formation of discrete dimers, Fig. 2a. These are formed by complexes of opposite chirality, held together by strong π–π stacking inter­actions *via* the planar tetra­aza­perylene moieties with *Cg*···*Cg* distances between 3.563 (3) and 3.837 (3) Å. The root mean square (rms) deviation from planarity for the tape moiety was calculated to be 0.0472 Å. The π–π stacking modes are very similar to earlier observed behavior of analogous complexes (Brietzke *et al.*, 2012). All arene rings of the tape ligand are involved in the π–π stacking, Fig. 2b. The dimer features an inter­planar tape separation of 3.33 Å. The Ru—N bond lengths formed by the tape and tmbpy ligands, Table 1, are very close to those of [Ru(L–L)tape]^2+^ (with L–L = bpy, dmbpy and dtbbpy), reported earlier (Brietzke *et al.*, 2012). The three-dimensional structure is characterized by the parallel lying tape moieties. These are divided by tmbpy moieties. As a consequence large space is available, which is filled with hexafluoridophosphate and aceto­nitrile. Lots of non-classical hydrogen bonds connect cations, anions and solvent molecules to stabilize the crystal packing, Fig 3. Furthermore, these are supported by weak C—H···π-ring and C—H···N, F inter­actions (Table 2) .

### 2. Synthesis and crystallization

The title compound was prepared as described earlier (Brietzke *et al.*, 2012). Crystals suitable for X-ray structure analysis were obtained by vapor diffusion of di­ethyl ether into a saturated aceto­nitrile solution of [Ru(tmbpy)_2_tape](PF_6_)_2_. Thus, the solution was filled into a test tube, placed into a di­ethyl ether containing bottle. Dark green crystals began to form at ambient temperature within one week.

### 3. Refinement

All hydrogen atoms were calculated in their expected positions and refined as riding atoms with *U*_iso_(H)=1.2*U*_eq_(C) and C—H distances of 0.93 Å for aromatic H atoms and with *U*_iso_(H)=1.5*U*_eq_(C) and distances of 0.96 Å for methyl hydrogen atoms. After unsuccessful attemps to model a disordered molecule of aceto­nitrile, the observed structure were modified by PLATON/SQUEEZE (Spek, 2009) to remove its contribution. PLATON/SQUEEZE calculated a solvent-accesible void volume in the unit cell of 305 Å^3^ (11.3 % of the total cell volume), corresponding to 44 electrons (residual electron density after the last refinement cycle) per unit cell. This number agrees with two molecules of aceto­nitrile (2 x 22 = 44). Four fluorines of one hexafluoridophosphate anion (on P1) are disordered over three sets of positions, with occupancies of 0.517 (3):0.244 (3):0.239 (3), respectively.

### Crystal data

**Table:**

[Ru(C_14_H_16_N_2_)_2_(C_16_H_8_N_4_)](PF_6_)_2_·3C_2_H_3_N	*Z* = 2
*M**_r_* = 1195.01	*F*(000) = 1216
Triclinic, *P*1	*D*_x_ = 1.471 Mg m^−^^3^
Hall symbol: -P 1	Mo *K*α radiation, λ = 0.71073 Å
*a* = 12.7485 (5) Å	Cell parameters from 17908 reflections
*b* = 13.6973 (7) Å	θ = 1.2–27.1°
*c* = 17.3623 (9) Å	µ = 0.44 mm^−^^1^
α = 105.786 (4)°	*T* = 210 K
β = 92.858 (4)°	Prism, dark green
γ = 110.436 (3)°	0.55 × 0.40 × 0.25 mm
*V* = 2698.3 (2) Å^3^	

### Data collection

**Table:**

Stoe IPDS-2 diffractometer	8918 independent reflections
Radiation source: fine-focus sealed tube	6396 reflections with *I* > 2σ(*I*)
Graphite monochromator	*R*_int_ = 0.044
ω scan	θ_max_ = 25.0°, θ_min_ = 1.7°
Absorption correction: integration (*X-RED*; Stoe & Cie, 2011)	*h* = −15→15
*T*_min_ = 0.758, *T*_max_ = 0.955	*k* = −16→16
17495 measured reflections	*l* = −20→20

### Refinement

**Table:**

Refinement on *F*^2^	Primary atom site location: structure-invariant direct methods
Least-squares matrix: full	Secondary atom site location: difference Fourier map
*R*[*F*^2^ > 2σ(*F*^2^)] = 0.047	Hydrogen site location: inferred from neighbouring sites
*wR*(*F*^2^) = 0.124	H-atom parameters constrained
*S* = 0.94	*w* = 1/[σ^2^(*F*_o_^2^) + (0.0767*P*)^2^] where *P* = (*F*_o_^2^ + 2*F*_c_^2^)/3
8918 reflections	(Δ/σ)_max_ = 0.048
743 parameters	Δρ_max_ = 0.51 e Å^−^^3^
363 restraints	Δρ_min_ = −0.54 e Å^−^^3^

### Special details

**Table:**

Geometry. All e.s.d.'s (except the e.s.d. in the dihedral angle between two l.s. planes) are estimated using the full covariance matrix. The cell e.s.d.'s are taken into account individually in the estimation of e.s.d.'s in distances, angles and torsion angles; correlations between e.s.d.'s in cell parameters are only used when they are defined by crystal symmetry. An approximate (isotropic) treatment of cell e.s.d.'s is used for estimating e.s.d.'s involving l.s. planes.
Refinement. Refinement of *F*^2^ against ALL reflections. The weighted *R*-factor *wR* and goodness of fit *S* are based on *F*^2^, conventional *R*-factors *R* are based on *F*, with *F* set to zero for negative *F*^2^. The threshold expression of *F*^2^ > 2σ(*F*^2^) is used only for calculating *R*-factors(gt) *etc*. and is not relevant to the choice of reflections for refinement. *R*-factors based on *F*^2^ are statistically about twice as large as those based on *F*, and *R*- factors based on ALL data will be even larger.

### Fractional atomic coordinates and isotropic or equivalent isotropic displacement parameters (Å^2^)

**Table:**

	*x*	*y*	*z*	*U*_iso_*/*U*_eq_	Occ. (<1)
C1	0.9535 (4)	0.5550 (3)	0.7768 (3)	0.0491 (10)	
H1	0.9615	0.5471	0.7227	0.059*	
C2	1.0166 (4)	0.6518 (3)	0.8328 (3)	0.0535 (11)	
H2	1.0652	0.7083	0.8165	0.064*	
C3	1.0691 (4)	0.7631 (4)	0.9812 (3)	0.0587 (12)	
H3	1.1193	0.8251	0.9717	0.070*	
C4	1.0525 (5)	0.7629 (4)	1.0582 (3)	0.0651 (13)	
H4	1.0935	0.8267	1.1002	0.078*	
C5	0.7638 (4)	0.4080 (4)	1.1288 (3)	0.0609 (12)	
H5	0.7596	0.4113	1.1828	0.073*	
C6	0.6955 (4)	0.3138 (4)	1.0720 (3)	0.0607 (12)	
H6	0.6458	0.2564	1.0869	0.073*	
C7	0.6375 (4)	0.2133 (4)	0.9220 (3)	0.0607 (12)	
H7	0.5850	0.1513	0.9301	0.073*	
C8	0.6524 (4)	0.2154 (3)	0.8455 (3)	0.0526 (11)	
H8	0.6092	0.1540	0.8023	0.063*	
C9	0.8701 (3)	0.4816 (3)	0.8733 (2)	0.0410 (9)	
C10	0.9343 (4)	0.5786 (3)	0.9364 (2)	0.0432 (9)	
C11	0.9233 (4)	0.5869 (3)	1.0184 (2)	0.0478 (10)	
C12	0.8452 (4)	0.4914 (3)	1.0381 (2)	0.0469 (10)	
C13	0.7788 (4)	0.3962 (3)	0.9733 (2)	0.0445 (9)	
C14	0.7897 (3)	0.3907 (3)	0.8918 (2)	0.0412 (9)	
C15	1.0090 (4)	0.6679 (3)	0.9168 (3)	0.0494 (10)	
C16	0.7017 (4)	0.3052 (4)	0.9898 (3)	0.0515 (10)	
C17	0.5981 (4)	0.4380 (3)	0.7574 (3)	0.0504 (10)	
H17	0.5880	0.4096	0.8006	0.060*	
C18	0.5422 (4)	0.5060 (4)	0.7492 (3)	0.0597 (12)	
C19	0.5588 (4)	0.5515 (4)	0.6870 (3)	0.0592 (12)	
C20	0.6286 (4)	0.5230 (4)	0.6332 (3)	0.0545 (11)	
H20	0.6410	0.5517	0.5902	0.065*	
C21	0.6795 (4)	0.4524 (3)	0.6434 (2)	0.0453 (10)	
C22	0.7530 (3)	0.4178 (3)	0.5882 (2)	0.0418 (9)	
C23	0.7676 (4)	0.4405 (3)	0.5163 (2)	0.0463 (10)	
H23	0.7321	0.4832	0.5016	0.056*	
C24	0.8338 (4)	0.4018 (3)	0.4652 (2)	0.0471 (10)	
C25	0.8860 (3)	0.3373 (3)	0.4896 (2)	0.0438 (9)	
C26	0.8688 (3)	0.3188 (3)	0.5624 (2)	0.0421 (9)	
H26	0.9051	0.2780	0.5790	0.051*	
C27	0.4646 (5)	0.5281 (5)	0.8090 (4)	0.0837 (17)	
H27A	0.4665	0.4915	0.8488	0.126*	
H27B	0.3885	0.5011	0.7808	0.126*	
H27C	0.4895	0.6055	0.8355	0.126*	
C28	0.5044 (6)	0.6280 (5)	0.6749 (3)	0.0866 (19)	
H28A	0.5374	0.6964	0.7176	0.130*	
H28B	0.4245	0.5963	0.6756	0.130*	
H28C	0.5164	0.6403	0.6236	0.130*	
C29	0.8467 (5)	0.4249 (4)	0.3863 (3)	0.0637 (13)	
H29A	0.8157	0.3573	0.3426	0.096*	
H29B	0.9256	0.4610	0.3846	0.096*	
H29C	0.8070	0.4713	0.3809	0.096*	
C30	0.9579 (4)	0.2884 (4)	0.4384 (3)	0.0611 (12)	
H30A	0.9115	0.2353	0.3891	0.092*	
H30B	0.9910	0.2533	0.4678	0.092*	
H30C	1.0169	0.3452	0.4258	0.092*	
C31	0.5323 (3)	0.1451 (3)	0.6379 (2)	0.0432 (9)	
H31	0.5073	0.2028	0.6455	0.052*	
C32	0.4521 (3)	0.0394 (3)	0.6086 (2)	0.0428 (9)	
C33	0.4911 (4)	−0.0465 (3)	0.5967 (2)	0.0468 (10)	
C34	0.6065 (4)	−0.0215 (3)	0.6128 (2)	0.0470 (10)	
H34	0.6337	−0.0778	0.6035	0.056*	
C35	0.6821 (3)	0.0867 (3)	0.6427 (2)	0.0406 (9)	
C36	0.8029 (3)	0.1188 (3)	0.6649 (2)	0.0424 (9)	
C37	0.8613 (4)	0.0500 (3)	0.6433 (3)	0.0508 (10)	
H37	0.8221	−0.0223	0.6114	0.061*	
C38	0.9769 (4)	0.0865 (3)	0.6681 (3)	0.0520 (11)	
C39	1.0332 (4)	0.1945 (3)	0.7174 (2)	0.0471 (10)	
C40	0.9713 (4)	0.2604 (3)	0.7350 (2)	0.0434 (9)	
H40	1.0093	0.3331	0.7665	0.052*	
C41	0.3296 (4)	0.0203 (4)	0.5928 (3)	0.0590 (11)	
H41A	0.3002	−0.0190	0.5366	0.089*	
H41B	0.3207	0.0895	0.6062	0.089*	
H41C	0.2890	−0.0217	0.6255	0.089*	
C42	0.4105 (4)	−0.1637 (3)	0.5686 (3)	0.0629 (13)	
H42A	0.3631	−0.1776	0.5190	0.094*	
H42B	0.3642	−0.1780	0.6092	0.094*	
H42C	0.4526	−0.2107	0.5595	0.094*	
C43	1.0396 (5)	0.0110 (4)	0.6420 (4)	0.0752 (15)	
H43A	1.0889	0.0354	0.6053	0.113*	
H43B	0.9861	−0.0621	0.6153	0.113*	
H43C	1.0837	0.0117	0.6887	0.113*	
C44	1.1565 (4)	0.2395 (4)	0.7511 (3)	0.0655 (13)	
H44A	1.1776	0.3125	0.7873	0.098*	
H44B	1.1999	0.2410	0.7075	0.098*	
H44C	1.1713	0.1939	0.7800	0.098*	
P1	0.28562 (13)	0.29009 (10)	0.53337 (8)	0.0656 (4)	
F1A	0.3747 (3)	0.4046 (3)	0.5343 (2)	0.1070 (13)	
F2A	0.1974 (3)	0.1762 (3)	0.5305 (2)	0.1152 (13)	
F3A	0.2296 (13)	0.3494 (9)	0.5977 (9)	0.187 (10)	0.517 (3)
F4A	0.2066 (10)	0.2982 (11)	0.4647 (8)	0.100 (5)	0.517 (3)
F5A	0.3413 (12)	0.2335 (8)	0.4672 (11)	0.168 (9)	0.517 (3)
F6A	0.3657 (11)	0.2851 (10)	0.6007 (9)	0.170 (10)	0.517 (3)
F3B	0.3496 (11)	0.2988 (11)	0.6149 (6)	0.052 (6)	0.244 (3)
F4B	0.2101 (12)	0.3466 (11)	0.5793 (11)	0.107 (11)	0.244 (3)
F5B	0.2216 (13)	0.2815 (13)	0.4509 (8)	0.082 (10)	0.244 (3)
F6B	0.3595 (13)	0.2331 (11)	0.4864 (9)	0.144 (15)	0.244 (3)
F3C	0.3232 (18)	0.3186 (15)	0.6257 (6)	0.096 (12)	0.239 (3)
F4C	0.1944 (12)	0.3408 (11)	0.550 (2)	0.150 (18)	0.239 (3)
F5C	0.250 (2)	0.2632 (16)	0.4400 (7)	0.19 (3)	0.239 (3)
F6C	0.3778 (14)	0.2404 (13)	0.516 (2)	0.167 (18)	0.239 (3)
P2	0.30495 (19)	−0.05892 (15)	0.82144 (11)	0.1025 (6)	
F7	0.3074 (8)	0.0519 (5)	0.8355 (7)	0.328 (7)	
F8	0.3046 (7)	−0.1780 (4)	0.8031 (5)	0.240 (4)	
F9	0.4063 (5)	−0.0332 (5)	0.7752 (3)	0.197 (3)	
F10	0.2122 (6)	−0.0902 (6)	0.8698 (4)	0.271 (5)	
F11	0.3925 (6)	−0.0232 (7)	0.8972 (3)	0.260 (5)	
F12	0.2236 (6)	−0.1051 (6)	0.7437 (4)	0.243 (4)	
N1	0.8782 (3)	0.4674 (2)	0.79498 (18)	0.0410 (7)	
N2	0.9818 (3)	0.6779 (3)	1.0784 (2)	0.0575 (10)	
N3	0.8370 (3)	0.4969 (3)	1.1151 (2)	0.0547 (9)	
N4	0.7285 (3)	0.3040 (2)	0.82864 (19)	0.0417 (7)	
N5	0.6659 (3)	0.4114 (2)	0.70604 (19)	0.0413 (8)	
N6	0.8028 (3)	0.3555 (2)	0.61199 (17)	0.0388 (7)	
N7	0.6437 (3)	0.1696 (2)	0.65597 (18)	0.0411 (8)	
N8	0.8593 (3)	0.2260 (2)	0.70947 (18)	0.0385 (7)	
Ru1	0.76414 (3)	0.32203 (2)	0.71845 (2)	0.03817 (11)	
C45	0.6212 (11)	−0.0934 (9)	0.9095 (7)	0.172 (4)	
H45A	0.6471	−0.0405	0.9626	0.258*	
H45B	0.6331	−0.1586	0.9102	0.258*	
H45C	0.5418	−0.1109	0.8942	0.258*	
C46	0.6826 (9)	−0.0492 (7)	0.8529 (6)	0.123 (3)	
N9	0.7363 (9)	−0.0128 (7)	0.8093 (6)	0.167 (3)	
C47	0.8808 (10)	0.8156 (10)	0.7475 (6)	0.172 (5)	
H47A	0.8579	0.8733	0.7417	0.259*	
H47B	0.8611	0.7587	0.6964	0.259*	
H47C	0.9613	0.8442	0.7647	0.259*	
C48	0.8237 (9)	0.7712 (9)	0.8072 (7)	0.142 (3)	
N10	0.7779 (10)	0.7379 (9)	0.8572 (6)	0.181 (4)	

### Atomic displacement parameters (Å^2^)

**Table:**

	*U*^11^	*U*^22^	*U*^33^	*U*^12^	*U*^13^	*U*^23^
C1	0.048 (3)	0.048 (2)	0.048 (2)	0.015 (2)	0.0124 (19)	0.0115 (18)
C2	0.050 (3)	0.043 (2)	0.062 (3)	0.008 (2)	0.011 (2)	0.019 (2)
C3	0.054 (3)	0.046 (2)	0.062 (3)	0.010 (2)	0.003 (2)	0.007 (2)
C4	0.063 (4)	0.050 (3)	0.063 (3)	0.013 (2)	0.004 (2)	0.000 (2)
C5	0.064 (4)	0.085 (3)	0.046 (2)	0.037 (3)	0.025 (2)	0.026 (2)
C6	0.057 (3)	0.072 (3)	0.058 (3)	0.024 (3)	0.023 (2)	0.026 (2)
C7	0.060 (3)	0.054 (3)	0.065 (3)	0.014 (2)	0.020 (2)	0.022 (2)
C8	0.049 (3)	0.049 (2)	0.055 (2)	0.014 (2)	0.014 (2)	0.0135 (19)
C9	0.037 (3)	0.0408 (19)	0.042 (2)	0.0148 (18)	0.0106 (17)	0.0069 (16)
C10	0.038 (3)	0.042 (2)	0.043 (2)	0.0150 (19)	0.0058 (17)	0.0043 (16)
C11	0.045 (3)	0.048 (2)	0.046 (2)	0.021 (2)	0.0094 (18)	0.0029 (18)
C12	0.043 (3)	0.055 (2)	0.043 (2)	0.023 (2)	0.0103 (18)	0.0109 (18)
C13	0.041 (3)	0.049 (2)	0.044 (2)	0.018 (2)	0.0143 (18)	0.0118 (17)
C14	0.038 (3)	0.042 (2)	0.044 (2)	0.0167 (19)	0.0121 (17)	0.0111 (17)
C15	0.045 (3)	0.043 (2)	0.054 (2)	0.015 (2)	0.0067 (19)	0.0073 (18)
C16	0.049 (3)	0.057 (2)	0.053 (2)	0.020 (2)	0.019 (2)	0.022 (2)
C17	0.047 (3)	0.051 (2)	0.056 (2)	0.024 (2)	0.016 (2)	0.0095 (19)
C18	0.054 (3)	0.059 (3)	0.062 (3)	0.032 (2)	0.011 (2)	−0.002 (2)
C19	0.058 (3)	0.058 (3)	0.061 (3)	0.034 (2)	0.002 (2)	0.002 (2)
C20	0.063 (3)	0.055 (2)	0.051 (2)	0.033 (2)	0.007 (2)	0.0107 (19)
C21	0.044 (3)	0.040 (2)	0.050 (2)	0.0174 (19)	0.0086 (18)	0.0060 (17)
C22	0.037 (3)	0.0349 (18)	0.047 (2)	0.0114 (18)	0.0036 (17)	0.0062 (16)
C23	0.050 (3)	0.039 (2)	0.046 (2)	0.017 (2)	0.0047 (19)	0.0070 (17)
C24	0.045 (3)	0.039 (2)	0.046 (2)	0.0083 (19)	0.0080 (18)	0.0062 (17)
C25	0.036 (3)	0.044 (2)	0.041 (2)	0.0104 (19)	0.0050 (17)	0.0031 (16)
C26	0.033 (3)	0.045 (2)	0.044 (2)	0.0157 (19)	0.0074 (17)	0.0063 (16)
C27	0.080 (4)	0.099 (4)	0.093 (4)	0.060 (4)	0.037 (3)	0.024 (3)
C28	0.108 (5)	0.097 (4)	0.078 (4)	0.077 (4)	0.017 (3)	0.012 (3)
C29	0.075 (4)	0.067 (3)	0.049 (2)	0.025 (3)	0.018 (2)	0.019 (2)
C30	0.060 (3)	0.074 (3)	0.050 (2)	0.033 (3)	0.019 (2)	0.010 (2)
C31	0.034 (3)	0.047 (2)	0.050 (2)	0.017 (2)	0.0038 (17)	0.0143 (17)
C32	0.032 (3)	0.049 (2)	0.044 (2)	0.0112 (19)	0.0049 (17)	0.0145 (17)
C33	0.040 (3)	0.046 (2)	0.045 (2)	0.011 (2)	0.0032 (18)	0.0077 (17)
C34	0.045 (3)	0.039 (2)	0.051 (2)	0.016 (2)	0.0014 (18)	0.0044 (17)
C35	0.035 (3)	0.043 (2)	0.040 (2)	0.0153 (19)	0.0063 (16)	0.0075 (16)
C36	0.038 (3)	0.040 (2)	0.045 (2)	0.0142 (19)	0.0034 (17)	0.0065 (16)
C37	0.044 (3)	0.042 (2)	0.061 (3)	0.020 (2)	0.006 (2)	0.0038 (18)
C38	0.042 (3)	0.051 (2)	0.064 (3)	0.022 (2)	0.011 (2)	0.013 (2)
C39	0.036 (3)	0.058 (2)	0.046 (2)	0.019 (2)	0.0031 (18)	0.0114 (18)
C40	0.037 (3)	0.048 (2)	0.040 (2)	0.0132 (19)	0.0027 (17)	0.0089 (16)
C41	0.042 (3)	0.061 (3)	0.077 (3)	0.017 (2)	0.011 (2)	0.028 (2)
C42	0.044 (3)	0.045 (2)	0.083 (3)	0.008 (2)	−0.001 (2)	0.007 (2)
C43	0.054 (4)	0.064 (3)	0.108 (4)	0.035 (3)	0.013 (3)	0.011 (3)
C44	0.048 (3)	0.075 (3)	0.068 (3)	0.027 (3)	0.001 (2)	0.009 (2)
P1	0.0775 (11)	0.0526 (7)	0.0667 (8)	0.0315 (7)	−0.0020 (7)	0.0117 (6)
F1A	0.116 (3)	0.0721 (19)	0.110 (3)	0.012 (2)	−0.037 (2)	0.0338 (18)
F2A	0.115 (4)	0.079 (2)	0.125 (3)	0.002 (2)	−0.015 (2)	0.042 (2)
F3A	0.36 (3)	0.203 (15)	0.115 (10)	0.214 (17)	0.126 (15)	0.073 (9)
F4A	0.073 (7)	0.140 (10)	0.102 (9)	0.040 (8)	0.001 (8)	0.065 (8)
F5A	0.143 (13)	0.093 (8)	0.27 (2)	0.070 (9)	0.101 (14)	0.013 (10)
F6A	0.178 (15)	0.080 (7)	0.223 (18)	0.018 (8)	−0.108 (14)	0.069 (9)
F3B	0.059 (12)	0.089 (12)	0.038 (7)	0.055 (11)	0.023 (7)	0.025 (7)
F4B	0.120 (19)	0.21 (3)	0.060 (10)	0.14 (2)	0.015 (10)	0.041 (12)
F5B	0.051 (14)	0.14 (2)	0.043 (10)	0.037 (16)	0.009 (10)	0.015 (11)
F6B	0.23 (3)	0.23 (3)	0.041 (9)	0.19 (3)	0.013 (11)	0.022 (11)
F3C	0.045 (11)	0.14 (2)	0.037 (7)	−0.019 (13)	0.015 (8)	0.004 (9)
F4C	0.068 (15)	0.078 (12)	0.31 (5)	0.027 (10)	−0.03 (2)	0.077 (19)
F5C	0.23 (5)	0.16 (3)	0.047 (12)	−0.09 (3)	−0.02 (2)	0.025 (15)
F6C	0.19 (4)	0.17 (3)	0.17 (3)	0.11 (3)	0.12 (3)	0.03 (2)
P2	0.1084 (17)	0.0854 (11)	0.0800 (11)	−0.0078 (10)	0.0051 (10)	0.0333 (9)
F7	0.346 (13)	0.100 (4)	0.573 (18)	0.103 (6)	0.239 (13)	0.106 (7)
F8	0.324 (11)	0.128 (5)	0.250 (8)	0.050 (6)	0.030 (7)	0.082 (5)
F9	0.185 (7)	0.242 (7)	0.157 (5)	0.050 (5)	0.078 (5)	0.084 (5)
F10	0.237 (9)	0.240 (7)	0.196 (6)	−0.064 (6)	0.122 (6)	0.039 (5)
F11	0.190 (7)	0.362 (11)	0.099 (4)	−0.014 (7)	−0.024 (4)	0.035 (5)
F12	0.233 (9)	0.265 (8)	0.159 (5)	0.029 (7)	−0.085 (5)	0.064 (5)
N1	0.040 (2)	0.0414 (17)	0.0426 (17)	0.0152 (16)	0.0121 (14)	0.0128 (14)
N2	0.057 (3)	0.054 (2)	0.048 (2)	0.017 (2)	0.0046 (17)	0.0003 (17)
N3	0.053 (3)	0.070 (2)	0.0403 (18)	0.027 (2)	0.0151 (16)	0.0100 (17)
N4	0.034 (2)	0.0397 (17)	0.0462 (18)	0.0119 (15)	0.0091 (14)	0.0082 (14)
N5	0.038 (2)	0.0381 (16)	0.0453 (18)	0.0160 (15)	0.0135 (15)	0.0064 (14)
N6	0.037 (2)	0.0370 (16)	0.0377 (16)	0.0159 (15)	0.0046 (14)	0.0027 (13)
N7	0.039 (2)	0.0395 (16)	0.0421 (17)	0.0149 (16)	0.0077 (14)	0.0077 (13)
N8	0.035 (2)	0.0394 (16)	0.0384 (16)	0.0135 (15)	0.0054 (14)	0.0085 (13)
Ru1	0.0355 (2)	0.03675 (17)	0.03886 (17)	0.01383 (14)	0.00804 (12)	0.00568 (12)
C45	0.202 (12)	0.162 (9)	0.177 (10)	0.084 (9)	0.077 (9)	0.064 (8)
C46	0.144 (9)	0.107 (6)	0.124 (7)	0.054 (6)	0.043 (6)	0.033 (5)
N9	0.178 (9)	0.152 (7)	0.188 (9)	0.057 (7)	0.074 (7)	0.079 (6)
C47	0.200 (12)	0.244 (12)	0.139 (8)	0.118 (10)	0.087 (8)	0.105 (9)
C48	0.124 (9)	0.152 (8)	0.149 (9)	0.073 (7)	0.009 (7)	0.019 (7)
N10	0.178 (10)	0.232 (11)	0.154 (8)	0.075 (8)	0.054 (7)	0.088 (8)

### Geometric parameters (Å, º)

**Table:**

C1—C2	1.351 (6)	C31—C32	1.384 (6)
C1—N1	1.375 (5)	C31—H31	0.9300
C1—H1	0.9300	C32—C33	1.401 (5)
C2—C15	1.428 (6)	C32—C41	1.487 (6)
C2—H2	0.9300	C33—C34	1.383 (6)
C3—C4	1.365 (7)	C33—C42	1.498 (6)
C3—C15	1.400 (6)	C34—C35	1.389 (5)
C3—H3	0.9300	C34—H34	0.9300
C4—N2	1.347 (6)	C35—N7	1.357 (5)
C4—H4	0.9300	C35—C36	1.446 (6)
C5—N3	1.342 (6)	C36—N8	1.364 (5)
C5—C6	1.354 (7)	C36—C37	1.382 (5)
C5—H5	0.9300	C37—C38	1.383 (6)
C6—C16	1.407 (6)	C37—H37	0.9300
C6—H6	0.9300	C38—C39	1.395 (6)
C7—C8	1.359 (6)	C38—C43	1.507 (6)
C7—C16	1.421 (6)	C39—C40	1.379 (5)
C7—H7	0.9300	C39—C44	1.493 (6)
C8—N4	1.379 (5)	C40—N8	1.346 (5)
C8—H8	0.9300	C40—H40	0.9300
C9—N1	1.335 (5)	C41—H41A	0.9600
C9—C10	1.412 (5)	C41—H41B	0.9600
C9—C14	1.437 (6)	C41—H41C	0.9600
C10—C15	1.402 (6)	C42—H42A	0.9600
C10—C11	1.415 (5)	C42—H42B	0.9600
C11—N2	1.327 (5)	C42—H42C	0.9600
C11—C12	1.479 (6)	C43—H43A	0.9600
C12—N3	1.328 (5)	C43—H43B	0.9600
C12—C13	1.421 (6)	C43—H43C	0.9600
C13—C16	1.404 (6)	C44—H44A	0.9600
C13—C14	1.413 (5)	C44—H44B	0.9600
C14—N4	1.334 (5)	C44—H44C	0.9600
C17—N5	1.340 (5)	P1—F6A	1.545 (7)
C17—C18	1.386 (6)	P1—F3C	1.550 (9)
C17—H17	0.9300	P1—F5A	1.551 (8)
C18—C19	1.376 (7)	P1—F6B	1.550 (9)
C18—C27	1.512 (6)	P1—F3B	1.551 (8)
C19—C20	1.395 (6)	P1—F4C	1.552 (9)
C19—C28	1.496 (6)	P1—F3A	1.552 (7)
C20—C21	1.381 (6)	P1—F6C	1.554 (9)
C20—H20	0.9300	P1—F4B	1.556 (8)
C21—N5	1.347 (5)	P1—F2A	1.559 (4)
C21—C22	1.475 (5)	P1—F5C	1.565 (9)
C22—N6	1.359 (5)	P1—F5B	1.567 (9)
C22—C23	1.372 (6)	P2—F7	1.460 (5)
C23—C24	1.382 (6)	P2—F10	1.493 (5)
C23—H23	0.9300	P2—F12	1.496 (5)
C24—C25	1.409 (6)	P2—F11	1.529 (6)
C24—C29	1.492 (6)	P2—F9	1.542 (5)
C25—C26	1.369 (5)	P2—F8	1.573 (6)
C25—C30	1.505 (5)	N1—Ru1	2.048 (3)
C26—N6	1.354 (5)	N4—Ru1	2.047 (3)
C26—H26	0.9300	N5—Ru1	2.074 (3)
C27—H27A	0.9600	N6—Ru1	2.065 (3)
C27—H27B	0.9600	N7—Ru1	2.063 (3)
C27—H27C	0.9600	N8—Ru1	2.061 (3)
C28—H28A	0.9600	C45—C46	1.420 (12)
C28—H28B	0.9600	C45—H45A	0.9600
C28—H28C	0.9600	C45—H45B	0.9600
C29—H29A	0.9600	C45—H45C	0.9600
C29—H29B	0.9600	C46—N9	1.143 (11)
C29—H29C	0.9600	C47—C48	1.444 (14)
C30—H30A	0.9600	C47—H47A	0.9600
C30—H30B	0.9600	C47—H47B	0.9600
C30—H30C	0.9600	C47—H47C	0.9600
C31—N7	1.339 (5)	C48—N10	1.176 (13)
			
C2—C1—N1	123.9 (4)	C37—C38—C39	118.2 (4)
C2—C1—H1	118.1	C37—C38—C43	120.5 (4)
N1—C1—H1	118.1	C39—C38—C43	121.4 (4)
C1—C2—C15	120.4 (4)	C40—C39—C38	118.0 (4)
C1—C2—H2	119.8	C40—C39—C44	119.9 (4)
C15—C2—H2	119.8	C38—C39—C44	122.1 (4)
C4—C3—C15	118.6 (4)	N8—C40—C39	124.0 (4)
C4—C3—H3	120.7	N8—C40—H40	118.0
C15—C3—H3	120.7	C39—C40—H40	118.0
N2—C4—C3	125.4 (4)	C32—C41—H41A	109.5
N2—C4—H4	117.3	C32—C41—H41B	109.5
C3—C4—H4	117.3	H41A—C41—H41B	109.5
N3—C5—C6	126.4 (4)	C32—C41—H41C	109.5
N3—C5—H5	116.8	H41A—C41—H41C	109.5
C6—C5—H5	116.8	H41B—C41—H41C	109.5
C5—C6—C16	118.2 (4)	C33—C42—H42A	109.5
C5—C6—H6	120.9	C33—C42—H42B	109.5
C16—C6—H6	120.9	H42A—C42—H42B	109.5
C8—C7—C16	120.4 (4)	C33—C42—H42C	109.5
C8—C7—H7	119.8	H42A—C42—H42C	109.5
C16—C7—H7	119.8	H42B—C42—H42C	109.5
C7—C8—N4	123.2 (4)	C38—C43—H43A	109.5
C7—C8—H8	118.4	C38—C43—H43B	109.5
N4—C8—H8	118.4	H43A—C43—H43B	109.5
N1—C9—C10	124.1 (4)	C38—C43—H43C	109.5
N1—C9—C14	116.0 (3)	H43A—C43—H43C	109.5
C10—C9—C14	119.9 (3)	H43B—C43—H43C	109.5
C15—C10—C9	119.0 (4)	C39—C44—H44A	109.5
C15—C10—C11	120.0 (4)	C39—C44—H44B	109.5
C9—C10—C11	121.0 (4)	H44A—C44—H44B	109.5
N2—C11—C10	121.9 (4)	C39—C44—H44C	109.5
N2—C11—C12	118.9 (4)	H44A—C44—H44C	109.5
C10—C11—C12	119.3 (3)	H44B—C44—H44C	109.5
N3—C12—C13	121.7 (4)	F6A—P1—F5A	90.7 (6)
N3—C12—C11	119.8 (4)	F6B—P1—F3B	90.0 (6)
C13—C12—C11	118.5 (3)	F3C—P1—F4C	90.5 (7)
C16—C13—C14	118.9 (4)	F6A—P1—F3A	91.0 (6)
C16—C13—C12	119.9 (4)	F5A—P1—F3A	177.9 (7)
C14—C13—C12	121.2 (4)	F3C—P1—F6C	89.6 (7)
N4—C14—C13	123.6 (4)	F4C—P1—F6C	179.4 (8)
N4—C14—C9	116.4 (3)	F6B—P1—F4B	179.1 (8)
C13—C14—C9	120.0 (4)	F3B—P1—F4B	90.8 (6)
C3—C15—C10	117.0 (4)	F6A—P1—F2A	90.2 (4)
C3—C15—C2	126.7 (4)	F3C—P1—F2A	90.9 (5)
C10—C15—C2	116.3 (4)	F5A—P1—F2A	90.3 (4)
C13—C16—C6	116.8 (4)	F6B—P1—F2A	89.9 (6)
C13—C16—C7	116.8 (4)	F3B—P1—F2A	90.0 (5)
C6—C16—C7	126.3 (4)	F4C—P1—F2A	90.1 (5)
N5—C17—C18	123.1 (4)	F3A—P1—F2A	91.0 (5)
N5—C17—H17	118.5	F6C—P1—F2A	90.5 (6)
C18—C17—H17	118.5	F4B—P1—F2A	89.8 (5)
C19—C18—C17	119.2 (4)	F3C—P1—F5C	178.7 (8)
C19—C18—C27	122.0 (4)	F4C—P1—F5C	90.0 (7)
C17—C18—C27	118.9 (5)	F6C—P1—F5C	89.9 (7)
C18—C19—C20	117.7 (4)	F2A—P1—F5C	90.3 (5)
C18—C19—C28	122.8 (4)	F6B—P1—F5B	89.8 (7)
C20—C19—C28	119.5 (5)	F3B—P1—F5B	179.7 (8)
C21—C20—C19	120.3 (4)	F4B—P1—F5B	89.4 (7)
C21—C20—H20	119.8	F2A—P1—F5B	90.2 (5)
C19—C20—H20	119.8	F7—P2—F10	94.1 (4)
N5—C21—C20	121.5 (4)	F7—P2—F12	93.9 (5)
N5—C21—C22	115.3 (3)	F10—P2—F12	92.6 (4)
C20—C21—C22	123.2 (4)	F7—P2—F11	92.3 (5)
N6—C22—C23	121.2 (3)	F10—P2—F11	89.6 (4)
N6—C22—C21	114.5 (3)	F12—P2—F11	173.2 (5)
C23—C22—C21	124.2 (4)	F7—P2—F9	90.4 (4)
C22—C23—C24	121.7 (4)	F10—P2—F9	174.2 (5)
C22—C23—H23	119.2	F12—P2—F9	90.7 (4)
C24—C23—H23	119.2	F11—P2—F9	86.5 (4)
C23—C24—C25	117.5 (4)	F7—P2—F8	177.6 (5)
C23—C24—C29	121.1 (4)	F10—P2—F8	88.1 (4)
C25—C24—C29	121.4 (4)	F12—P2—F8	85.0 (4)
C26—C25—C24	117.7 (3)	F11—P2—F8	88.6 (4)
C26—C25—C30	119.8 (4)	F9—P2—F8	87.5 (4)
C24—C25—C30	122.5 (4)	C9—N1—C1	116.2 (3)
N6—C26—C25	124.9 (4)	C9—N1—Ru1	114.1 (3)
N6—C26—H26	117.6	C1—N1—Ru1	129.3 (3)
C25—C26—H26	117.6	C11—N2—C4	117.2 (4)
C18—C27—H27A	109.5	C12—N3—C5	116.9 (4)
C18—C27—H27B	109.5	C14—N4—C8	117.0 (3)
H27A—C27—H27B	109.5	C14—N4—Ru1	114.0 (3)
C18—C27—H27C	109.5	C8—N4—Ru1	128.9 (3)
H27A—C27—H27C	109.5	C17—N5—C21	118.1 (3)
H27B—C27—H27C	109.5	C17—N5—Ru1	126.1 (3)
C19—C28—H28A	109.5	C21—N5—Ru1	115.6 (2)
C19—C28—H28B	109.5	C26—N6—C22	117.0 (3)
H28A—C28—H28B	109.5	C26—N6—Ru1	127.0 (3)
C19—C28—H28C	109.5	C22—N6—Ru1	116.0 (2)
H28A—C28—H28C	109.5	C31—N7—C35	118.8 (3)
H28B—C28—H28C	109.5	C31—N7—Ru1	126.1 (2)
C24—C29—H29A	109.5	C35—N7—Ru1	114.5 (3)
C24—C29—H29B	109.5	C40—N8—C36	118.1 (3)
H29A—C29—H29B	109.5	C40—N8—Ru1	126.8 (2)
C24—C29—H29C	109.5	C36—N8—Ru1	114.7 (3)
H29A—C29—H29C	109.5	N4—Ru1—N1	79.34 (13)
H29B—C29—H29C	109.5	N4—Ru1—N8	91.00 (12)
C25—C30—H30A	109.5	N1—Ru1—N8	100.20 (12)
C25—C30—H30B	109.5	N4—Ru1—N7	92.60 (13)
H30A—C30—H30B	109.5	N1—Ru1—N7	171.90 (12)
C25—C30—H30C	109.5	N8—Ru1—N7	79.05 (13)
H30A—C30—H30C	109.5	N4—Ru1—N6	174.66 (12)
H30B—C30—H30C	109.5	N1—Ru1—N6	96.31 (12)
N7—C31—C32	124.1 (3)	N8—Ru1—N6	92.82 (12)
N7—C31—H31	118.0	N7—Ru1—N6	91.79 (12)
C32—C31—H31	118.0	N4—Ru1—N5	98.07 (12)
C31—C32—C33	117.3 (4)	N1—Ru1—N5	85.26 (12)
C31—C32—C41	120.2 (4)	N8—Ru1—N5	170.18 (12)
C33—C32—C41	122.5 (4)	N7—Ru1—N5	96.70 (13)
C34—C33—C32	118.8 (4)	N6—Ru1—N5	78.38 (12)
C34—C33—C42	119.9 (4)	C46—C45—H45A	109.5
C32—C33—C42	121.3 (4)	C46—C45—H45B	109.5
C33—C34—C35	120.7 (4)	H45A—C45—H45B	109.5
C33—C34—H34	119.7	C46—C45—H45C	109.5
C35—C34—H34	119.7	H45A—C45—H45C	109.5
N7—C35—C34	120.3 (4)	H45B—C45—H45C	109.5
N7—C35—C36	115.8 (3)	N9—C46—C45	177.0 (13)
C34—C35—C36	123.8 (3)	C48—C47—H47A	109.5
N8—C36—C37	120.3 (4)	C48—C47—H47B	109.5
N8—C36—C35	115.0 (3)	H47A—C47—H47B	109.5
C37—C36—C35	124.7 (4)	C48—C47—H47C	109.5
C36—C37—C38	121.3 (4)	H47A—C47—H47C	109.5
C36—C37—H37	119.4	H47B—C47—H47C	109.5
C38—C37—H37	119.4	N10—C48—C47	178.2 (13)
			
N1—C1—C2—C15	0.9 (7)	C30—C25—C26—N6	−177.7 (4)
C15—C3—C4—N2	−0.3 (8)	N7—C31—C32—C33	−0.6 (6)
N3—C5—C6—C16	1.4 (8)	N7—C31—C32—C41	178.0 (4)
C16—C7—C8—N4	0.0 (7)	C31—C32—C33—C34	−1.5 (6)
N1—C9—C10—C15	2.3 (6)	C41—C32—C33—C34	179.9 (4)
C14—C9—C10—C15	−177.5 (4)	C31—C32—C33—C42	177.4 (4)
N1—C9—C10—C11	−178.5 (4)	C41—C32—C33—C42	−1.2 (6)
C14—C9—C10—C11	1.7 (6)	C32—C33—C34—C35	2.1 (6)
C15—C10—C11—N2	0.2 (6)	C42—C33—C34—C35	−176.8 (4)
C9—C10—C11—N2	−179.1 (4)	C33—C34—C35—N7	−0.5 (6)
C15—C10—C11—C12	−179.2 (4)	C33—C34—C35—C36	176.7 (4)
C9—C10—C11—C12	1.6 (6)	N7—C35—C36—N8	11.4 (5)
N2—C11—C12—N3	−1.8 (6)	C34—C35—C36—N8	−165.9 (4)
C10—C11—C12—N3	177.6 (4)	N7—C35—C36—C37	−167.8 (4)
N2—C11—C12—C13	177.0 (4)	C34—C35—C36—C37	14.9 (6)
C10—C11—C12—C13	−3.6 (6)	N8—C36—C37—C38	2.1 (6)
N3—C12—C13—C16	0.4 (6)	C35—C36—C37—C38	−178.8 (4)
C11—C12—C13—C16	−178.3 (4)	C36—C37—C38—C39	1.4 (7)
N3—C12—C13—C14	−178.9 (4)	C36—C37—C38—C43	−178.3 (4)
C11—C12—C13—C14	2.4 (6)	C37—C38—C39—C40	−3.3 (6)
C16—C13—C14—N4	1.6 (6)	C43—C38—C39—C40	176.3 (4)
C12—C13—C14—N4	−179.1 (4)	C37—C38—C39—C44	176.6 (4)
C16—C13—C14—C9	−178.4 (4)	C43—C38—C39—C44	−3.8 (7)
C12—C13—C14—C9	0.9 (6)	C38—C39—C40—N8	2.0 (6)
N1—C9—C14—N4	−2.9 (5)	C44—C39—C40—N8	−177.9 (4)
C10—C9—C14—N4	176.9 (3)	C10—C9—N1—C1	−1.0 (6)
N1—C9—C14—C13	177.2 (3)	C14—C9—N1—C1	178.8 (3)
C10—C9—C14—C13	−3.0 (6)	C10—C9—N1—Ru1	−175.3 (3)
C4—C3—C15—C10	0.6 (7)	C14—C9—N1—Ru1	4.5 (4)
C4—C3—C15—C2	−178.7 (4)	C2—C1—N1—C9	−0.7 (6)
C9—C10—C15—C3	178.7 (4)	C2—C1—N1—Ru1	172.7 (3)
C11—C10—C15—C3	−0.6 (6)	C10—C11—N2—C4	0.2 (6)
C9—C10—C15—C2	−1.9 (6)	C12—C11—N2—C4	179.6 (4)
C11—C10—C15—C2	178.9 (4)	C3—C4—N2—C11	−0.1 (8)
C1—C2—C15—C3	179.8 (5)	C13—C12—N3—C5	0.6 (6)
C1—C2—C15—C10	0.4 (6)	C11—C12—N3—C5	179.3 (4)
C14—C13—C16—C6	178.8 (4)	C6—C5—N3—C12	−1.6 (7)
C12—C13—C16—C6	−0.5 (6)	C13—C14—N4—C8	−1.1 (6)
C14—C13—C16—C7	−1.2 (6)	C9—C14—N4—C8	178.9 (3)
C12—C13—C16—C7	179.5 (4)	C13—C14—N4—Ru1	179.8 (3)
C5—C6—C16—C13	−0.3 (6)	C9—C14—N4—Ru1	−0.2 (4)
C5—C6—C16—C7	179.7 (5)	C7—C8—N4—C14	0.3 (6)
C8—C7—C16—C13	0.5 (7)	C7—C8—N4—Ru1	179.3 (3)
C8—C7—C16—C6	−179.5 (4)	C18—C17—N5—C21	−0.3 (6)
N5—C17—C18—C19	−2.0 (7)	C18—C17—N5—Ru1	174.2 (3)
N5—C17—C18—C27	178.1 (5)	C20—C21—N5—C17	2.1 (6)
C17—C18—C19—C20	2.4 (7)	C22—C21—N5—C17	−178.6 (4)
C27—C18—C19—C20	−177.7 (5)	C20—C21—N5—Ru1	−173.0 (3)
C17—C18—C19—C28	−178.4 (5)	C22—C21—N5—Ru1	6.2 (4)
C27—C18—C19—C28	1.6 (8)	C25—C26—N6—C22	−2.0 (6)
C18—C19—C20—C21	−0.7 (7)	C25—C26—N6—Ru1	175.4 (3)
C28—C19—C20—C21	−179.9 (5)	C23—C22—N6—C26	1.2 (5)
C19—C20—C21—N5	−1.7 (7)	C21—C22—N6—C26	178.2 (3)
C19—C20—C21—C22	179.2 (4)	C23—C22—N6—Ru1	−176.5 (3)
N5—C21—C22—N6	−4.5 (5)	C21—C22—N6—Ru1	0.5 (4)
C20—C21—C22—N6	174.8 (4)	C32—C31—N7—C35	2.1 (6)
N5—C21—C22—C23	172.5 (4)	C32—C31—N7—Ru1	−168.2 (3)
C20—C21—C22—C23	−8.3 (7)	C34—C35—N7—C31	−1.5 (5)
N6—C22—C23—C24	−0.3 (6)	C36—C35—N7—C31	−178.9 (3)
C21—C22—C23—C24	−177.1 (4)	C34—C35—N7—Ru1	169.9 (3)
C22—C23—C24—C25	0.1 (6)	C36—C35—N7—Ru1	−7.5 (4)
C22—C23—C24—C29	178.5 (4)	C39—C40—N8—C36	1.5 (6)
C23—C24—C25—C26	−0.8 (6)	C39—C40—N8—Ru1	−170.6 (3)
C29—C24—C25—C26	−179.2 (4)	C37—C36—N8—C40	−3.5 (6)
C23—C24—C25—C30	178.7 (4)	C35—C36—N8—C40	177.3 (3)
C29—C24—C25—C30	0.3 (6)	C37—C36—N8—Ru1	169.6 (3)
C24—C25—C26—N6	1.8 (6)	C35—C36—N8—Ru1	−9.6 (4)

### Hydrogen-bond geometry (Å, º)

*Cg*1, *Cg*2 and *Cg*3 denote the centroids of the N7/C31–C35, N8/C36–C40 and N6/C22–C26 rings, respectively.

**Table:**

*D*—H···*A*	*D*—H	H···*A*	*D*···*A*	*D*—H···*A*
C2—H2···F10^i^	0.93	2.61	3.402 (7)	143
C3—H3···F10^i^	0.93	2.50	3.318 (8)	147
C6—H6···F8^ii^	0.93	2.58	3.218 (8)	126
C8—H8···N7	0.93	2.65	3.166 (5)	116
C20—H20···F1*A*^iii^	0.93	2.41	3.320 (6)	166
C20—H20···F5*C*^iii^	0.93	2.64	3.411 (15)	141
C23—H23···F1*A*^iii^	0.93	2.55	3.479 (5)	177
C26—H26···N8	0.93	2.59	3.138 (5)	118
C31—H31···F6*A*	0.93	2.63	3.461 (13)	150
C34—H34···F5*A*^iv^	0.93	2.29	3.170 (8)	158
C34—H34···F6*B*^iv^	0.93	2.30	3.129 (11)	148
C34—H34···F6*C*^iv^	0.93	2.55	3.295 (19)	137
C45—H45*A*···F11^ii^	0.96	2.49	3.343 (12)	148
C45—H45*C*···F11	0.96	2.58	3.388 (15)	142
C47—H47*A*···N9^v^	0.96	2.66	3.452 (14)	140
C47—H47*B*···F5*B*^iii^	0.96	2.55	3.343 (16)	140
C47—H47*B*···F5*C*^iii^	0.96	2.59	3.293 (15)	131
C8—H8···*Cg*1	0.93	2.92	3.711 (5)	144
C26—H26···*Cg*2	0.93	2.90	3.708 (4)	146
C42—H42*A*···*Cg*3^vi^	0.96	2.79	3.339 (5)	117

Symmetry codes: (i) *x*+1, *y*+1, *z*; (ii) −*x*+1, −*y*, −*z*+2; (iii) −*x*+1, −*y*+1, −*z*+1; (iv) −*x*+1, −*y*, −*z*+1; (v) *x*, *y*+1, *z*; (vi) −*x*+1, −*y*+2, −*z*+1.
